# Reproducible Bradycardia After Flumazenil Reversal of Midazolam-Propofol Intravenous Sedation in a Patient With Klinefelter Syndrome: A Case Report

**DOI:** 10.7759/cureus.110275

**Published:** 2026-06-04

**Authors:** Katsuhiro Matsumoto, Shigeharu Jinno, Shigeru Maeda, Tsuneto Owatari

**Affiliations:** 1 Department of Dental Anesthesiology, Graduate School of Medical and Dental Sciences, Institute of Science Tokyo, Tokyo, JPN; 2 Dentistry, Shimada Ryoiku Medical Center for Challenged Children, Tokyo, JPN; 3 Dental Center of the Medically Compromised Patient, Kyushu Dental University, Fukuoka, JPN

**Keywords:** bradycardia, dental treatment, flumazenil, heart rate, intravenous sedation, klinefelter syndrome, midazolam, propofol

## Abstract

Klinefelter syndrome (KS) is the most common sex chromosome aneuploidy in males and is associated with metabolic, cardiovascular, and neurodevelopmental features. Some individuals with KS have also been reported to exhibit altered heart rate (HR) regulation, although cardiovascular responses associated with intravenous sedation (IV-S) have not been well documented.

We report a 14-year-old boy with KS, intellectual disability, and autism spectrum disorder who underwent multiple dental treatments under IV-S with midazolam and propofol. During three consecutive sessions at approximately two-week intervals, flumazenil administration at the end of each session was followed by reproducible declines in HR of 13-21 beats per minute, while blood pressure and oxygen saturation showed no marked deterioration. The post-flumazenil HR was lower than both the pre-sedation baseline HR and the lowest HR recorded during dental treatment in each session. The patient had no history of structural heart disease, and post-procedure chest radiography and transthoracic echocardiogram showed no significant abnormalities. These observations raise the possibility that the abrupt pharmacologic transition associated with flumazenil reversal, together with KS-related vulnerability in HR regulation, contributed to the reproducible post-reversal bradycardia. Careful monitoring during reversal and emergence may be warranted when flumazenil is used in patients with KS.

## Introduction

Klinefelter syndrome (KS) is the most common sex chromosome aneuploidy in males. It is associated with hypogonadism, neurocognitive and psychosocial features, and an increased risk of metabolic and cardiovascular abnormalities [1]. Beyond these widely recognized features, clinical studies have suggested that a subset of individuals with KS shows altered heart rate (HR) regulation, including chronotropic incompetence, defined as an impaired ability to increase HR appropriately in response to cardiovascular demand, with possible involvement of autonomic regulation relevant to HR control [2,3].

In patients with KS whose neurodevelopmental features limit cooperation, dental treatment may require anesthetic management, including intravenous sedation (IV-S) [4,5]. For dental treatment under IV-S, midazolam and propofol are commonly used in combination [6]. Flumazenil, a competitive antagonist of benzodiazepine receptors, is used to reverse sedation caused by midazolam during IV-S [7]. Although these agents can influence autonomic balance and hemodynamics [8,9], cardiovascular changes during IV-S in patients with KS have not been well documented. We report a patient with KS who experienced reproducible bradycardia immediately after flumazenil administration at the end of midazolam-propofol IV-S during three consecutive dental procedures. Written informed consent was obtained from the patient’s parents for publication of this case report and accompanying images.

## Case presentation

A 14-year-old boy, 172 cm in height and weighing 64.4 kg, had been diagnosed with KS (47, XXY karyotype) at five years of age, along with intellectual disability and autism spectrum disorder. Accompanied by his parents, the patient presented to our hospital with oral pain. Intraoral examination revealed dental caries in several teeth requiring dental treatment. There was no history of cardiovascular disease. A preoperative 12-lead electrocardiogram (ECG) showed normal sinus rhythm (HR 68 bpm (beats per minute)) with incomplete right bundle branch block. Because of behavioral difficulties, routine dental procedures while awake could not be performed; therefore, his dental procedures were scheduled for IV-S over multiple visits.

**Figure 1:**
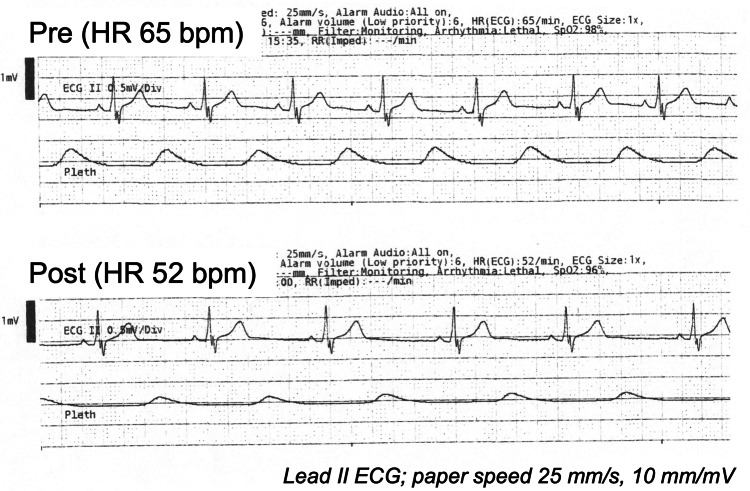
Electrocardiographic findings before and after flumazenil administration in Session 3 Lead II electrocardiogram (ECG) tracings were obtained from the intravenous sedation session in which the post-reversal heart rate (HR) decline was first recognized: immediately before flumazenil administration (Pre; HR 65 bpm) and within approximately 1 min after administration (Post; HR 52 bpm). Sinus rhythm was observed in both displayed tracings.

During one of these IV-S sessions, within approximately 1 min after flumazenil administration at the end of the procedure, we observed a clear decrease in HR (from 65 to 52 bpm). In contrast, blood pressure (BP) and percutaneous oxygen saturation (SpO₂) remained stable, and the ECG showed sinus rhythm without ectopic beats or conduction abnormalities (Figure 1). HR appeared to increase with eye opening. This post-reversal HR decline prompted us to review the anesthetic records from that session and the two preceding sessions at our hospital, all of which were performed with the same IV-S protocol.

In this report, we describe three consecutive IV-S sessions. Sedation was induced with a single intravenous bolus of midazolam (Session 1: 4 mg; Session 2: 3 mg; Session 3: 4 mg), without additional bolus administration. Propofol was then infused continuously, starting at 6 mg/kg/h and later reduced to 5 mg/kg/h in Session 1, starting at 7 mg/kg/h and titrated between 3 and 7 mg/kg/h in Session 2, and starting at 7 mg/kg/h and reduced to 5 mg/kg/h during the procedure in Session 3; dosing was adjusted clinically to permit completion of the planned dental treatment while minimizing patient movement and resistance to treatment and maintaining spontaneous ventilation. The propofol infusion was discontinued at the end of dental treatment. The first dose of flumazenil was administered intravenously approximately 2-3 min after discontinuation of the propofol infusion in each session. Flumazenil was given in accordance with standard dosing recommendations: an initial dose of 0.2 mg was administered slowly, followed by additional 0.1 mg increments at 1-min intervals as needed until the desired level of consciousness was achieved. The total dose did not exceed 0.5 mg in any session [7]. ECG, noninvasive BP, SpO₂, and end-tidal carbon dioxide concentration were continuously monitored throughout each procedure and emergence. Supplemental oxygen at 3 L/min was administered via nasal cannula throughout IV-S. Table 1 summarizes the total doses of midazolam, propofol, and flumazenil and the duration of IV-S for each session.

**Table 1:** Sedation parameters and hemodynamic variables across three sessions of midazolam–propofol intravenous sedation. Baseline HR indicates the value recorded before initiation of IV-S. Mean HR during dental treatment was calculated from values recorded during the dental treatment phase before discontinuation of propofol. HR range during dental treatment indicates the lowest and highest HR values recorded during the same phase. For HR before/after FLM, the pre-FLM value indicates the HR recorded before flumazenil administration, and the post-FLM value indicates the lowest HR recorded after flumazenil administration. For BP and SpO₂ before/after FLM, the values represent measurements recorded before and after flumazenil administration. MDZ, midazolam; FLM, flumazenil; HR, heart rate; BP, blood pressure; SpO₂, percutaneous oxygen saturation; IV-S, intravenous sedation.

Session	Dental procedure	IV-S duration (min)	Total doses (mg)			Baseline HR (bpm)	Mean HR during dental treatment (bpm)	HR range during dental treatment (bpm)	HR before/after FLM (bpm)	BP before/after FLM (mmHg)	SpO_2_ before/after FLM (%)
			MDZ	Propofol	FLM						
1	Root canal obturation for a tooth with apical periodontitis	73	4	354	0.4	68	68.1	63–75	60 → 44	121/61 → 124/79	99 → 97
2	Impression taking for fabrication of a crown	71	3	280	0.2	72	69.6	61–83	66 → 45	103/50 → 112/60	99 → 98
3	Cementation of the crown	28	4	129	0.5	75	69.8	62–73	65 → 52	101/57 → 94/49	98 → 97

**Figure 2:**
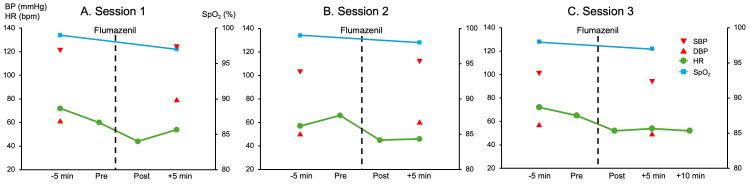
Vital-sign changes around flumazenil administration during the three intravenous sedation sessions (A) Session 1, (B) Session 2, and (C) Session 3. HR is shown at peri-reversal time points: 5 min before flumazenil administration, immediately before administration (Pre), shortly after administration (Post), and 5 min after administration; in Session 3, HR was also recorded 10 min after administration. SBP, DBP, and SpO₂ are shown 5 min before and 5 min after flumazenil administration. The dashed vertical line indicates flumazenil administration. In all three sessions, HR decreased shortly after flumazenil administration, whereas SBP, DBP, and SpO₂ showed no marked deterioration. HR, heart rate; SBP, systolic blood pressure; DBP, diastolic blood pressure; SpO₂, percutaneous oxygen saturation.

The three sessions were conducted approximately two weeks apart. In Session 1, root canal obturation was performed for a tooth with apical periodontitis. Session 2 involved impression-taking for the fabrication of a crown for the same tooth, and Session 3 involved cementation of that crown. No local anesthetic was administered in any of the three sessions because these procedures were not expected to cause clinically significant pain. During the dental treatment phase, HR varied within the ranges shown in Table 1. No respiratory or hemodynamic adverse events were observed in any of the three sessions. After flumazenil administration at the end of dental treatment, HR decreased from 60 to 44 bpm in Session 1, from 66 to 45 bpm in Session 2, and from 65 to 52 bpm in Session 3 (Table 1, Figure 2). In each session, the post-flumazenil HR was lower than both the pre-sedation baseline HR and the lowest HR recorded during dental treatment (Table 1). BP and SpO₂ showed no marked change after flumazenil administration in any session (Table 1, Figure 2).

After each procedure, the patient was observed in the recovery area for less than 30 min before discharge. No concerning signs or symptoms were noted during this period. Prolonged observation in the clinic was difficult because the patient had difficulty remaining in the recovery area for an extended period, owing to his intellectual disability. He was discharged in the care of both parents, who were instructed to continue close observation at home, particularly regarding his breathing and responsiveness, and to contact our hospital or seek medical attention at a nearby medical facility if any concerning changes occurred. No adverse events were reported after discharge. To evaluate for potential underlying structural heart abnormalities, chest X-ray and transthoracic echocardiography were performed after these procedures and showed no significant abnormalities.

## Discussion

We describe a patient with KS who repeatedly experienced bradycardia during emergence from midazolam-propofol IV-S, reversed with flumazenil. Although a modest reduction in pulse rate has been observed after administration of flumazenil alone in healthy volunteers [10], bradycardia associated with flumazenil occurs in less than 1% of cases [7]. HR usually remains stable during recovery from midazolam sedation, whereas it may be lower after propofol sedation [8]. The patient consistently exhibited a marked decline in HR immediately after flumazenil administration, while BP and SpO₂ remained within acceptable ranges. This response, observed across three independent sedation sessions, is unlikely to reflect a typical recovery-related change in HR. Because local anesthesia was not used in any of the three sessions, a procedure- or pain-related vagal response was considered as an alternative explanation [11]. However, this mechanism appeared less likely because the HR decline occurred immediately after flumazenil administration at the end of the procedures rather than during active dental manipulation, and a similar pattern was reproducibly observed across sessions despite differences in the procedures. Cardiac arrhythmia or structural heart disease was also considered; however, ECG findings and subsequent cardiac evaluation were unremarkable. We therefore considered whether the pharmacologic transition associated with flumazenil reversal at emergence, together with patient-specific factors affecting HR regulation, could have contributed to the reproducible decline in HR.

During each session, IV-S was performed using a combination of midazolam and propofol. Propofol has been associated with parasympathetic predominance and can lower HR, whereas midazolam has been associated with a shift toward sympathetic predominance and may maintain or slightly raise HR [8]. Together, these opposing autonomic influences may have produced a relatively stable HR before flumazenil reversal. At emergence, flumazenil rapidly antagonized midazolam’s central benzodiazepine effects [7,9], producing a pharmacologic transition from combined midazolam-propofol sedation to a state in which residual propofol effects may have persisted. This transition may have shifted autonomic balance toward parasympathetic predominance. Because HR is regulated by reciprocal sympathetic and parasympathetic inputs to the sinoatrial node [12,13], such an abrupt shift could make a bradycardic response apparent. However, this pharmacodynamic transition alone would not be expected to cause a marked HR decline in most patients. The reproducibility and magnitude of the HR decline across the three sessions therefore suggest that patient-specific factors affecting HR regulation may have modified the patient’s response to this pharmacologic transition.

The patient’s KS may be relevant when considering such patient-specific factors. Previous studies have reported chronotropic incompetence, defined as an impaired ability to increase HR appropriately in response to physiologic demand, in some patients with KS [2,3]. In the present case, however, HR varied during the dental procedures. This finding did not clearly suggest a markedly impaired chronotropic response. Nevertheless, no formal chronotropic assessment was performed. Autonomic and adrenergic alterations that may influence chronotropic activity and HR responses have also been described in KS [14], although these alterations were not formally assessed in our patient. Taken together, the previously reported KS-associated findings provide a physiologic context for the possibility that KS-related susceptibility in HR regulation may have modified the patient’s HR response during the abrupt post-flumazenil pharmacologic transition and may thereby have contributed to the reproducible HR decline.

This case emphasizes practical considerations for recovery management during midazolam-propofol IV-S in patients with KS. Respiratory or hemodynamic adverse events may occur during emergence and recovery after IV-S [15,16]. A previous report also described bradycardia accompanied by hypotension and impaired consciousness after flumazenil administration during recovery from procedural sedation [17]. In our patient, the bradycardic response was not accompanied by other clinically apparent adverse signs or symptoms. Nevertheless, when an immediate post-reversal HR decline is observed, reassessment of respiratory status, hemodynamics, and level of consciousness may be reasonable, because the finding may represent an isolated transient response or a sign of broader recovery-phase instability. If a marked immediate post-reversal HR decline is recognized, subsequent sedation plans may be reconsidered to minimize abrupt pharmacologic reversal when possible and to allow safer recovery observation.

In addition to monitoring the immediate post-reversal transition, patients should be observed for a sufficient period after flumazenil administration because re-sedation or respiratory depression may occur [18,19]. In the present case, the patient remained in the recovery area for less than 30 min after each procedure because prolonged observation in the clinic was difficult owing to his intellectual disability. Although no adverse events were reported after discharge, a longer in-clinic observation period would be preferable when feasible. When prolonged in-clinic observation is difficult, careful discharge planning should ensure that caregivers understand the need for continued observation after discharge.

## Conclusions

We report a patient with KS who exhibited a reproducible, immediate bradycardic response after flumazenil reversal during emergence from midazolam-propofol IV-S. This pattern suggests that the abrupt pharmacologic transition associated with flumazenil reversal may have unmasked clinically apparent bradycardia in the setting of KS-related vulnerability in HR regulation. Although the underlying mechanism remains uncertain, careful monitoring during reversal and emergence may be warranted when flumazenil is used in patients with KS.
